# Crystal structure of bis­[(1-ammonio-1-phosphono­eth­yl)phospho­nato]tetra­aqua­cadmium dihydrate: a powder X-ray diffraction study

**DOI:** 10.1107/S2056989015004028

**Published:** 2015-03-07

**Authors:** Mwaffak Rukiah, Thaer Assaad

**Affiliations:** a Department of Chemistry, Atomic Energy Commission of Syria (AECS), PO Box 6091, Damascus, Syrian Arab Republic; b Radioisotope Department, Atomic Energy Commission of Syria (AECS), PO Box 6091, Damascus, Syrian Arab Republic

**Keywords:** crystal structure, bis­phospho­nate complexes, complexes, cadmium, powder diffraction, octa­hedral coordination, zwitterion, hydrogen bonding

## Abstract

In the title compound, [Cd*L*_2_(H_2_O)_4_]·2H_2_O [*L* = (1-ammonio-1-phosphono­eth­yl)phospho­nate, C_2_H_8_NO_6_P_2_^−^], the Cd^II^ ion is situated on an inversion centre being coordinated by four aqua mol­ecules in the equatorial plane and two phosphonate O atoms from two deprotonated *L* ligands in the axial positions in a distorted octa­hedral geometry. The asymmetric unit contains one-half of the complex mol­ecule and one lattice water mol­ecule. The ligand *L* exists in a zwitterionic form, with a positive charge on the NH_3_ group and a negative charge on the O atom of the non-coordinating phospho­nate group, and with an intra­molecular O—H⋯O inter­action forming an *S*(6) ring motif and two intra­molecular N—H⋯O inter­actions each generating an *S*(5) ring motif. In the crystal, N—H⋯O and O—H⋯O hydrogen bonds link the complex mol­ecules into a three-dimensional network in which the voids of 38 Å^3^ are filled with ordered lattice water mol­ecules, which are also involved in O—H⋯O hydrogen bonding.

## Abstract 2

In the compound [Cd*L*_2_(H_2_O)_4_]·2H_2_O [*L* = (1-ammonio-1-phosphono­eth­yl)phospho­nate, C_2_H_8_NO_6_P_2_^−^], the Cd^II^ ion is situated on an inversion centre being coordinated by four aqua mol­ecules in the equatorial plane and two deprotonated phospho­nate O atoms from two *L* ligands in the axial positions in a distorted octa­hedral geometry. Each ligand *L* exists in a zwitterionic form, and with an intra­molecular O—H⋯O inter­action forming an *S*(6) ring motif and two intra­molecular N—H⋯O inter­actions each generating an *S*(5) ring motif. In the crystal, N—H⋯O and O—H⋯O hydrogen bonds link complex mol­ecules into a three-dimensional network with voids of 38 Å^3^ filled with ordered lattice water mol­ecules, which are also involved in O—H⋯O hydrogen bonding.

## Chemical context   

As a result of of their inhibitory effect on bone resorption, various types of bis­phospho­nates are used in the treatment of bone metastasis and several bone disorders such as Paget’s disease, and for the prevention of osteoporosis in post-menopausal women (Shaw & Bishop, 2005 ▸). Drugs prepared on the basis of bis­phospho­nates are highly efficient as a regulator of calcium metabolism and the immune response; they are used as anti-neoplastic, anti-inflammatory and anti­viral agents, drugs with analgesic effect and, as a component of toothpastes, bi­phospho­nates prevent the formation of tartar (Matkovskaya *et al.*, 2001 ▸). Organic di­phospho­nic acids are potentially very powerful chelating agents, used in metal extractions and have been tested by the pharmaceutical industry for use as efficient drugs preventing calcification and inhibiting bone resorption (Matczak-Jon & Videnova-Adrabińska, 2005 ▸). Di­phospho­nic acids and their metal complexes are used in the treatment of Paget’s disease, osteoporosis and tumoral osteolysis (Szabo *et al.*, 2002 ▸). However, it is still not clearly understood why small structural modification of bis­phospho­nates may lead to extensive alterations in their physicochemical, biological and toxicol­ogical characteristics (Matczak-Jon & Videnova-Adrabińska, 2005 ▸). Therefore, the structure determination of bis­phos­phon­ates is very important in order to understand the influence of structural modifications on their complex-forming abilities and physiological activities.
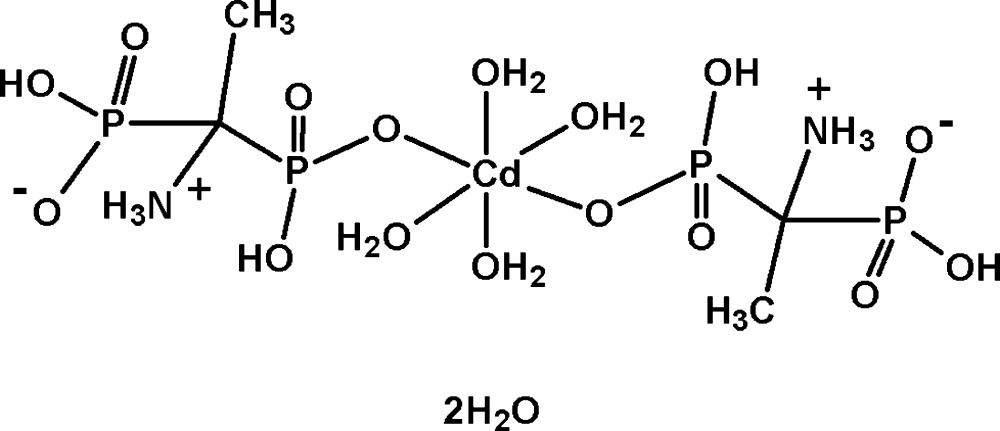


## Structural commentary   

**Figure 1:**
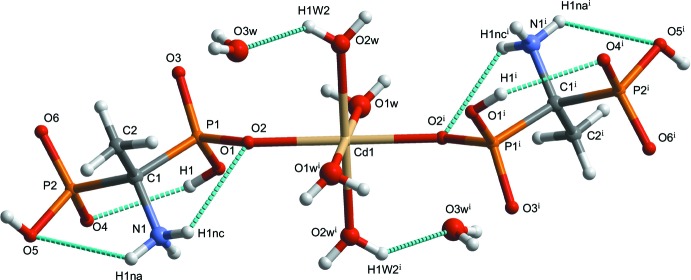
The mol­ecular structure of (I), showing the atom-labelling scheme [symmetry code: (i) −*x* + 1, −*y* + 1, −*z* + 1]. Displacement spheres are drawn at the 50% probability level. H atoms are represented as small spheres of arbitrary radii. Dotted lines denote hydrogen bonds.

The asymmetric unit of the title compound, (I) (Fig. 1 ▸), contains one half of the complex mol­ecule [Cd*L*_2_(H_2_O)_4_] [*L* = (1-ammonio-1-phosphono­eth­yl)phospho­nate] and one lattice water mol­ecule. All bond lengths and angles in (I) are normal and correspond to those observed in bis­phospho­nate complexes with transition metals (Shkol’nikova *et al.*, 1991 ▸; Sergienko *et al.*, 1997 ▸, 1999 ▸; Yin *et al.*, 2005 ▸; Li *et al.*, 2006 ▸; Li & Sun, 2007 ▸; Lin *et al.*, 2007 ▸; Xiang *et al.*, 2007 ▸; Dudko *et al.*, 2009 ▸, 2010 ▸; Bon *et al.*, 2010 ▸; Tsaryk *et al.*, 2010 ▸, 2011 ▸). The Cd^II^ atom occupies a special position on an inversion centre and shows a slightly distorted octa­hedral coordination environment formed by two phospho­nic O atoms in *trans* positions and four aqua O atoms in the equatorial plane. The distorted octa­hedral coordination polyhedron is slightly compressed in the axial direction; the Cd1—O2 bond length is 0.1 Å shorter than the Cd1—O1*W* and Cd1—O2*W* bonds. The values of the axial O—Cd—O angles are in the range 80.1 (4)–99.9 (4)°, indicating a significant deviation from ideal values. The ligand *L* exists in a zwitterionic form, with a positive charge on the NH_3_ group and a negative charge on the O atom of the non-coordinating phosphonate group, and with an intra­molecular O—H⋯O inter­action forming an *S*(6) ring motif and two intra­molecular N—H⋯O inter­actions each generating an *S*(5) ring motif (Table 1 ▸).

## Supra­molecular features   

**Figure 2:**
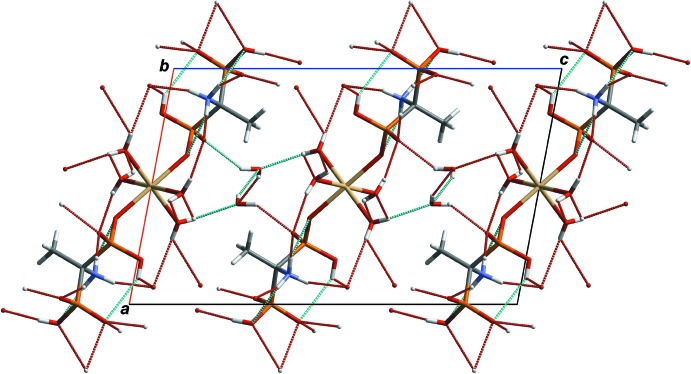
A portion of the crystal packing viewed down the *b* axis. Dashed lines denote hydrogen bonds.

The crystal packing is illustrated in Fig. 2 ▸ as a projection of the unit cell along the *b* axis. Inter­molecular N—H⋯O and O—H⋯O hydrogen bonds (Table 1 ▸) link complex mol­ecules into a three-dimensional network in which the voids of 38 Å^3^ are filled with ordered lattice water mol­ecules, which are also involved in O—H⋯O hydrogen bonding (Table 1 ▸ and Fig. 2 ▸).

## Synthesis and crystallization   

All reactions and manipulations were carried out in air with reagent grade solvents. 1-Amino­ethane-1,1-diyldi­phospho­nic acid was prepared according to the literature method of Rukiah & Assaad (2013 ▸). The title compound (I) was prepared by adding 10 ml of an 0.01 *M* CdCl_2_ aqueous solution to 10 ml of a 0.02 *M* water solution of 1-amino­ethane-1,1-diyldi­phospho­nic acid. A crude product was obtained after two weeks of slow evaporation of the resulted solution. It was further purified by recrystallization from ethanol and water (1:3 *v*/*v*) at 273 K to produce the title compound (I) (white powder; m.p. > 623 K) in 80% yield. The IR spectrum was recorded on a Jasco FT–IR 300E instrument and the ^1^H and ^13^C{^1^H} NMR spectra were recorded on a Bruker Bio spin 400 spectrometer.

**Spectroscopic data for (I):**

^1^H NMR (D_2_O, p.p.m.): δ 1.67 (*t*, 3H, CH_3_, *J* = 14 Hz). ^13^C{^1^H} NMR (D_2_O, p.p.m.): δ 20.5 (1C; CH_3_), 54.7 (1C; C—CH_3_). ^31^P{^1^H} NMR (D_2_O, p.p.m.): δ 13.61(2P; P—OH). IR (KBr, ν cm^−1^): 3446.2 (NH_3_), 2351.5 (POH), 1605.0 (O=P—O—H).

## Refinement   

Crystal data, data collection and structure refinement details are summarized in Table 2 ▸. Compound (I) has a tendency to crystallize in the form of a very fine white powder. Since no single crystals of sufficient size and quality could be obtained, a crystal structure determination from laboratory powder X-ray diffraction data was performed. The powder sample was ground slightly in a mortar, loaded into two Mylar foils and fixed onto the sample holder with a mask of suitable inter­nal diameter (8.0 mm). The powder X-ray diffraction data were collected at room temperature with a STOE transmission STADI-P diffractometer using CuK_α1_ radiation (λ= 1.54060 Å) selected with an incident-beam curved-crystal Ge(111) monochromator with a linear position-sensitive detector (PSD). The pattern was scanned over the angular range 6.0–90.0° (2θ). For pattern indexing, extraction of the peak positions was carried out with the program *WinPLOTR* (Roisnel & Rodríguez-Carvajal, 2001 ▸). Pattern indexing was performed with the program *DICVOL4.0* (Boultif & Louër, 2004 ▸). The first 20 intense peaks of the powder pattern were indexed completely on the basis of a monoclinic cell. The figures of merit (de Wolff *et al.*, 1968 ▸; Smith & Snyder, 1979 ▸) are sufficiently acceptable to support the obtained indexing results [*M*(20) = 37.1, *F*(20) = 78.5(0.0061, 42)]. The best estimated monoclinic space group was *P*2_1_/*c*.

The powder pattern was subsequently refined with cell and resolution constraints (Le Bail *et al.*, 1988 ▸) using the profile-matching option of the program *FULLPROF* (Rodríguez-Carvajal, 2001 ▸). The number of mol­ecules per unit cell was estimated to be *Z* = 2. The initial crystal structure was determined by direct methods using the program *EXPO2014* (Altomare *et al.*, 2013 ▸). The model found by this program was introduced into the program *GSAS* (Larson & Von Dreele, 2004 ▸) implemented in *EXPGUI* (Toby, 2001 ▸) for Rietveld refinement. The background was refined using a shifted Chebyshev polynomial with 20 coefficients. The effect of the asymmetry of the low-order peaks was corrected using a pseudo-Voigt description of the peak shape (Thompson *et al.*, 1987 ▸), angle-dependent asymmetry with axial divergence (Finger *et al.*, 1994 ▸) and microstrain broadening (Stephens, 1999 ▸). Two asymmetry parameters of this function, *S*/*L* and *D*/*L*, were both fixed at 0.0225 during this refinement. Intensities were corrected for absorption effects with a function for a plate sample in transmission geometry with μ·*d* value of 0.7585 (μ is the absorption coefficient and *d* is the sample thickness). These μ·*d* values were determined experimentally. The preferred orientation was modelled with 12 coefficients using a spherical harmonics correction (Von Dreele, 1997 ▸) of intensities. The use of the preferred orientation correction leads to a better mol­ecular geometry with better agreement factors. The value of obtained median texture index (1.0654) and the agreement factors in the refinement without texture correction (*R*_p_ = 0.053, *R*_wp_ = 0.073, *R*_exp _ = 0.025, *R*(*F* ^2^) = 0.011009 and χ^2^ = 8.940) indicate that the preferred orientation improvement of the refinement is considerable.

**Figure 3:**
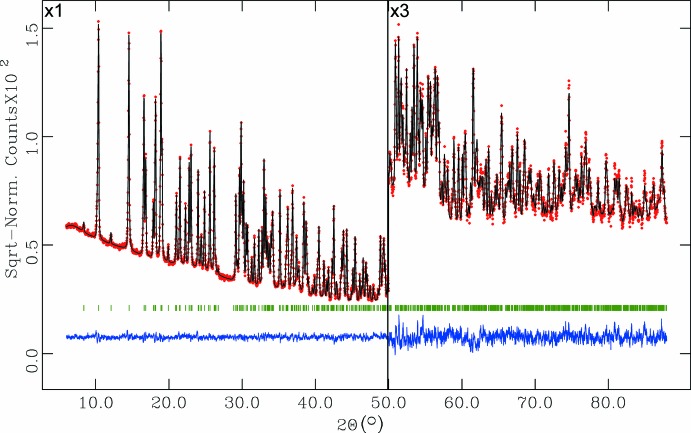
The final Rietveld plot for (I). Experimental intensities are indicated by dots, and the best-fit calculated (upper trace) and difference (lower trace) patterns are shown as solid lines. The vertical bars indicate the calculated positions of the Bragg peaks.

Before the final refinement, the H atoms of the CH_3_ and NH_3_ groups were introduced on the basis of geometrical arguments. The hy­droxy and water H atoms were located using the program *HYDROGEN* (Nardelli, 1999 ▸) implemented in *WinGX* (Farrugia, 2012 ▸). The coordinates of all H atoms were refined with very strict soft restraints on bond lengths and angle until a suitable geometry was obtained, after that they were fixed in the final stage of the refinement. Four restraints for the central carbon atom (C—CH_3_, C—NH_3_ and two C—PO_3_) on bond lengths were applied to normal values for these bonds. The final refinement cycles were performed varying isotropic displacement parameters for Cd and water O atoms, and fixed isotropic displacement parameters for P, C, N,O and H atoms. The final Rietveld plot is shown in Fig. 3 ▸.

## Supplementary Material

Crystal structure: contains datablock(s) I. DOI: 10.1107/S2056989015004028/cv5484sup1.cif

Rietveld powder data: contains datablock(s) I. DOI: 10.1107/S2056989015004028/cv5484Isup2.rtv

CCDC reference: 1051338

Additional supporting information:  crystallographic information; 3D view; checkCIF report

## Figures and Tables

**Table 1:** Hydrogen-bond geometry (, )

*D*H*A*	*D*H	H*A*	*D* *A*	*D*H*A*
O1H1O4	0.84	2.44	3.196(13)	151
N1H1NAO6^i^	0.87	2.07	2.828(13)	146
N1H1NBO4^ii^	0.88	2.16	2.872(15)	137
N1H1NCO3^i^	0.86	1.99	2.796(14)	156
O1*W*H1*W*1O2*W* ^i^	0.82	2.39	2.987(13)	131
O5H5O6^iii^	0.84	1.79	2.551(12)	150
O1*W*H2*W*1O3^i^	0.82	1.96	2.758(15)	162
O2*W*H2*W*2O4^iv^	0.82	2.35	3.141(15)	162
O3WH1*W*3O3W^v^	0.82	2.56	3.346(13)	160
O3WH2*W*3O3^vi^	0.82	2.13	2.833(14)	143

Symmetry codes: (i) 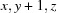; (ii) 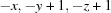; (iii) 
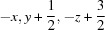
; (iv) 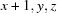; (v) 
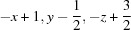
; (vi) 
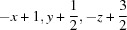
.

**Table 2:** Experimental details

Crystal data	
Chemical formula	[Cd(C_2_H_8_NO_6_P_2_)_2_(H_2_O)_4_]2H_2_O
*M*_r_	628.57
Crystal system, space group	Monoclinic, *P*2_1_/*c*
Temperature (K)	298
*a*, *b*, *c* ()	10.69424(12), 5.61453(5), 17.2737(2)
()	100.7029(8)
*V* (^3^)	1019.12(2)
*Z*	2
Radiation type	Cu *K*_1_, = 1.5406
(mm^1^)	12.41
Specimen shape, size (mm)	Flat sheet, 8 8

Data collection	
Diffractometer	Stoe transmission STADI-P
Specimen mounting	Powder loaded into two Mylar foils
Data collection mode	Transmission
Scan method	Step
Absorption correction	For a cylinder mounted on the axis [*GSAS* (Larson Von Dreele, 2004 ▸) absorption/surface roughness correction: function No. 4, flat-plate transmission absorption correction, terms = 0.75850]
*T*_min_, *T*_max_	0.195, 0.310
2 values ()	2_min_ = 6.00 2_max_ = 89.98 2_step_ = 0.02

Refinement	
*R* factors and goodness of fit	*R*_p_ = 0.029, *R*_wp_ = 0.039, *R*_exp_ = 0.025, *R*(*F* ^2^) = 0.04534, ^2^ = 2.624
No. of data points	4100
No. of parameters	133
No. of restraints	4
H-atom treatment	H-atom parameters not refined

Computer programs: *WinXPOW* (Stoe Cie, 1999 ▸), *GSAS* (Larson Von Dreele, 2004 ▸), *EXPO2014* (Altomare *et al.*, 2013 ▸), *Mercury* (Macrae *et al.*, 2006 ▸) and *publCIF* (Westrip, 2010 ▸).

## supplementary crystallographic information

### Crystal data

**Table:**

[Cd(C_2_H_8_NO_6_P_2_)_2_(H_2_O)_4_]·2H_2_O	*Z* = 2
*M**_r_* = 628.57	*F*(000) = 636
Monoclinic, *P*2_1_/*c*	*D*_x_ = 2.048 Mg m^−^^3^
Hall symbol: -P 2ybc	Cu *K*α_1_ radiation, λ = 1.5406 Å
*a* = 10.69424 (12) Å	µ = 12.41 mm^−^^1^
*b* = 5.61453 (5) Å	*T* = 298 K
*c* = 17.2737 (2) Å	Particle morphology: fine powder
β = 100.7029 (8)°	white
*V* = 1019.12 (2) Å^3^	flat sheet, 8 × 8 mm

### Data collection

**Table:**

Stoe transmission STADI-P diffractometer	Scan method: step
Radiation source: sealed X-ray tube	Absorption correction: for a cylinder mounted on the φ axis [*GSAS* (Larson & Von Dreele, 2004) absorption/surface roughness correction: function No. 4, flat-plate transmission absorption correction, terms = 0.75850]
Ge 111 monochromator	*T*_min_ = 0.195, *T*_max_ = 0.310
Specimen mounting: Powder loaded into two Mylar foils	2θ_min_ = 6.00°, 2θ_max_ = 89.98°, 2θ_step_ = 0.02°
Data collection mode: transmission	

### Refinement

**Table:**

Refinement on *I*_net_	Profile function: CW Profile function number 4 with 21 terms Pseudovoigt profile coefficients as parameterized in (Thompson *et al.*, 1987). Asymmetry correction of (Finger *et al.*, 1994). Microstrain broadening by (Stephens, 1999). #1(GU) = 0.000 #2(GV) = 0.000 #3(GW) = 7.136 #4(GP) = 0.000 #5(LX) = 2.421 #6(ptec) = 0.11 #7(trns) = 0.00 #8(shft) = 0.0000 #9(sfec) = 0.00 #10(S/L) = 0.0225 #11(H/L) = 0.0225 #12(eta) = 0.6026 #13(S400 ) = 1.2E-02 #14(S040 ) = 1.0E-01 #15(S004 ) = 1.2E-03 #16(S220 ) = 3.3E-02 #17(S202 ) = 6.7E-03 #18(S022 ) = 1.7E-02 #19(S301 ) = 1.1E-02 #20(S103 ) = 2.3E-03 #21(S121 ) = 1.5E-02 Peak tails are ignored where the intensity is below 0.0010 times the peak Aniso. broadening axis 0.0 0.0 1.0
Least-squares matrix: full	133 parameters
*R*_p_ = 0.029	4 restraints
*R*_wp_ = 0.039	H-atom parameters not refined
*R*_exp_ = 0.025	Weighting scheme based on measured s.u.'s
*R*(*F*^2^) = 0.04534	(Δ/σ)_max_ = 0.03
χ^2^ = 2.624	Background function: GSAS Background function number 1 with 20 terms. Shifted Chebyshev function of 1st kind 1: 1216.00 2: -1325.95 3: 695.908 4: -224.478 5: 51.5854 6: -12.9254 7: 7.98937 8: -13.4593 9: 4.35490 10: 32.6578 11: -32.8988 12: -6.52632 13: -18.8133 14: 23.4504 15: -2.70081 16: -0.874623 17: -28.0163 18: 27.3423 19: 2.28961 20: -11.2631
4100 data points	

### Fractional atomic coordinates and isotropic or equivalent isotropic displacement parameters (Å^2^)

**Table:**

	*x*	*y*	*z*	*U*_iso_*/*U*_eq_	
C1	0.1555 (5)	0.3764 (12)	0.6381 (5)	0.015*	
C2	0.2285 (11)	0.4133 (17)	0.7222 (5)	0.015*	
H2a	0.17609	0.50488	0.75213	0.02*	
H2b	0.3063	0.50177	0.72104	0.02*	
H2c	0.24919	0.26144	0.74755	0.02*	
N1	0.1274 (11)	0.6170 (16)	0.6028 (7)	0.02*	
H1na	0.07228	0.68814	0.62561	0.025*	
H1nb	0.09382	0.59823	0.55236	0.025*	
H1nc	0.19624	0.69829	0.60678	0.025*	
P1	0.2594 (5)	0.2361 (7)	0.5774 (3)	0.01*	
O1	0.1897 (8)	0.2047 (13)	0.4907 (6)	0.01*	
H1	0.11151	0.1887	0.48952	0.015*	
O2	0.3659 (9)	0.4155 (15)	0.5771 (6)	0.01*	
O3	0.3024 (8)	−0.0094 (16)	0.6124 (6)	0.01*	
P2	0.0019 (4)	0.2252 (6)	0.6342 (3)	0.01*	
O4	−0.0728 (9)	0.1904 (14)	0.5539 (6)	0.01*	
O5	−0.0807 (8)	0.4058 (11)	0.6745 (5)	0.01*	
H5	−0.06622	0.38276	0.7235	0.03*	
O6	0.0255 (7)	−0.0022 (16)	0.6785 (5)	0.01*	
Cd1	0.5	0.5	0.5	0.0093 (7)*	
O1W	0.5302 (11)	0.8211 (15)	0.5815 (7)	0.029 (4)*	
H1W1	0.56	0.91732	0.55443	0.03*	
H2W1	0.47216	0.89419	0.59649	0.03*	
O2W	0.6537 (10)	0.2995 (14)	0.5866 (6)	0.020 (3)*	
H1W2	0.63758	0.22791	0.62489	0.03*	
H2W2	0.72024	0.23865	0.57871	0.03*	
O3w	0.5739 (11)	0.3949 (14)	0.7310 (6)	0.028 (3)*	
H1W3	0.54032	0.26314	0.72841	0.03*	
H2W3	0.5752	0.43812	0.77663	0.03*	

### Geometric parameters (Å, º)

**Table:**

C1—C2	1.530 (12)	P2—O5	1.588 (7)
C1—N1	1.490 (12)	P2—O6	1.486 (9)
C1—P1	1.840 (9)	O5—H5	0.842
C1—P2	1.839 (7)	Cd1—O2	2.183 (8)
C2—H2a	0.976	Cd1—O2^i^	2.183 (8)
C2—H2b	0.972	Cd1—O1W	2.274 (9)
C2—H2c	0.965	Cd1—O1W^i^	2.274 (9)
N1—H1na	0.865	Cd1—O2W	2.300 (10)
N1—H1nb	0.885	Cd1—O2W^i^	2.300 (10)
N1—H1nc	0.858	O1W—H1W1	0.817
P1—O1	1.555 (11)	O1W—H2W1	0.824
P1—O2	1.521 (9)	O2W—H1W2	0.820
P1—O3	1.541 (9)	O2W—H2W2	0.823
O1—H1	0.838	O3w—H1W3	0.820
P2—O4	1.480 (10)	O3w—H2W3	0.823
			
C2—C1—N1	107.2 (7)	C1—P2—O6	108.2 (5)
C2—C1—P1	110.1 (6)	O4—P2—O5	104.3 (5)
C2—C1—P2	113.1 (7)	O4—P2—O6	112.3 (6)
N1—C1—P1	104.6 (5)	O5—P2—O6	112.2 (5)
N1—C1—P2	107.0 (6)	P2—O5—H5	109.2
P1—C1—P2	114.3 (4)	O2—Cd1—O2^i^	180.0
C1—C2—H2a	109.4	O2—Cd1—O1W	80.1 (4)
C1—C2—H2b	109.6	O2—Cd1—O1W^i^	99.9 (4)
C1—C2—H2c	110.1	O2—Cd1—O2W	88.2 (3)
H2a—C2—H2b	108.6	O2—Cd1—O2W^i^	91.8 (3)
H2a—C2—H2c	109.4	O2^i^—Cd1—O1W	99.9 (4)
H2b—C2—H2c	109.7	O2^i^—Cd1—O1W^i^	80.1 (4)
C1—N1—H1na	109.5	O2^i^—Cd1—O2W	91.8 (3)
C1—N1—H1nb	108.0	O2^i^—Cd1—O2W^i^	88.2 (3)
C1—N1—H1nc	110.1	O1W—Cd1—O1W^i^	180.0
H1na—N1—H1nb	108.5	O1W—Cd1—O2W	89.0 (4)
H1na—N1—H1nc	111.4	O1W—Cd1—O2W^i^	91.0 (4)
H1nb—N1—H1nc	109.1	O1W^i^—Cd1—O2W	91.0 (4)
C1—P1—O1	111.5 (6)	O1W^i^—Cd1—O2W^i^	89.0 (4)
C1—P1—O2	104.5 (5)	O2W—Cd1—O2W^i^	180.0
C1—P1—O3	109.1 (4)	Cd1—O1W—H1W1	101.2
O1—P1—O2	107.2 (5)	Cd1—O1W—H2W1	124.1
O1—P1—O3	109.4 (5)	H1W1—O1W—H2W1	104.3
O2—P1—O3	115.1 (6)	Cd1—O2W—H1W2	122.2
P1—O1—H1	109.4	Cd1—O2W—H2W2	129.1
P1—O2—Cd1	136.4 (6)	H1W2—O2W—H2W2	104.3
C1—P2—O4	114.8 (5)	H1W3—O3w—H2W3	104.3
C1—P2—O5	104.8 (4)		
			
O1W—Cd1—O2—P1	168.8 (9)	O1—P1—C1—C2	−177.9 (6)
O2W—Cd1—O2—P1	−101.9 (8)	O2—P1—C1—C2	−62.3 (7)
O1W^i^—Cd1—O2—P1	−11.2 (9)	O3—P1—C1—C2	61.3 (7)
O2W^i^—Cd1—O2—P1	78.1 (8)	O4—P2—C1—P1	−54.2 (6)
O3—P1—O2—Cd1	84.9 (9)	O5—P2—C1—P1	−168.0 (5)
C1—P1—O2—Cd1	−155.4 (7)	O6—P2—C1—P1	72.1 (6)
O1—P1—O2—Cd1	−37.0 (9)	O4—P2—C1—N1	61.1 (8)
O1—P1—C1—P2	53.6 (6)	O5—P2—C1—N1	−52.7 (8)
O2—P1—C1—P2	169.2 (5)	O6—P2—C1—N1	−172.6 (7)
O3—P1—C1—P2	−67.2 (6)	O4—P2—C1—C2	178.9 (6)
O1—P1—C1—N1	−63.1 (7)	O5—P2—C1—C2	65.0 (7)
O2—P1—C1—N1	52.5 (8)	O6—P2—C1—C2	−54.9 (7)
O3—P1—C1—N1	176.1 (7)		

Symmetry code: (i) −*x*+1, −*y*+1, −*z*+1.

### Hydrogen-bond geometry (Å, º)

**Table:**

*D*—H···*A*	*D*—H	H···*A*	*D*···*A*	*D*—H···*A*
O1—H1···O4	0.84	2.44	3.196 (13)	151
O1—H1···O4^ii^	0.84	2.27	2.592 (12)	103
N1—H1NA···O5	0.87	2.53	2.986 (14)	114
N1—H1NA···O6^iii^	0.87	2.07	2.828 (13)	146
N1—H1NB···O4^iv^	0.88	2.16	2.872 (15)	137
N1—H1NC···O2	0.86	2.53	2.899 (15)	107
N1—H1NC···O3^iii^	0.86	1.99	2.796 (14)	156
O1*W*—H1*W*1···O2*W*^iii^	0.82	2.39	2.987 (13)	131
O5—H5···O6^v^	0.84	1.79	2.551 (12)	150
O1*W*—H2*W*1···O3^iii^	0.82	1.96	2.758 (15)	162
O2*W*—H1*W*2···O3W	0.82	2.27	2.833 (15)	126
O2*W*—H2*W*2···O4^vi^	0.82	2.35	3.141 (15)	162
O3W—H1*W*3···O3W^vii^	0.82	2.56	3.346 (13)	160
O3W—H2*W*3···O3^viii^	0.82	2.13	2.833 (14)	143

Symmetry codes: (ii) −*x*, −*y*, −*z*+1; (iii) *x*, *y*+1, *z*; (iv) −*x*, −*y*+1, −*z*+1; (v) −*x*, *y*+1/2, −*z*+3/2; (vi) *x*+1, *y*, *z*; (vii) −*x*+1, *y*−1/2, −*z*+3/2; (viii) −*x*+1, *y*+1/2, −*z*+3/2.
